# Factors Influencing Symptoms of Depression, Anxiety and Stress in Patients With Haemophilia

**DOI:** 10.1111/hae.70079

**Published:** 2025-06-27

**Authors:** Alexander Schmidt, Fabian Tomschi, Pia Möllers, Marius Brühl, Heinrich Richter, Johannes Oldenburg, Andreas Christian Strauss, Thomas Hilberg

**Affiliations:** ^1^ Department of Sports Medicine University of Wuppertal Wuppertal Germany; ^2^ Department of Orthopaedics and Trauma Surgery University Hospital Bonn Bonn Germany; ^3^ Haemophilia Centre Muenster Muenster Germany; ^4^ Institute for Experimental Haematology and Transfusion Medicine University Hospital Bonn Bonn Germany

**Keywords:** anxiety, depression, haemophilia, haemophilic arthropathy, pain, stress

## Abstract

**Introduction:**

Patients with haemophilia (PwH) often suffer from psychological symptoms such as depression or anxiety. To date, uncertainty exists about the determinants predicting worse psychological outcomes. Therefore, this study aimed to investigate the extent of depressive, anxiety and stress‐related symptoms in PwH compared to the healthy population and determine the impact of disease‐specific and arthropathy‐related parameters.

**Methods:**

Levels of depression, anxiety, stress and overall emotional distress were queried in a total of 379 PwH and 271 healthy controls by handing out the Depression, Anxiety and Stress Scale 21. In addition, disease‐specific variables (e.g., type, severity, viral infections), pain intensity (NRS from 0 to 10), pain persistence (Likert‐scale from 1 to 6), pain sensitivity (pressure pain thresholds [PPT]) and the orthopaedic joint score (Haemophilia Joint Health Score v2.1; HJHS) were assessed to analyse associations with psychological symptoms.

**Results:**

PwH had higher scores for depression, anxiety, stress and overall emotional distress compared to the healthy cohort. Regarding disease‐specific outcomes, only PwH with hepatitis or HIV showed higher scores for depression (hepatitis, *p* = 0.020), stress (hepatitis, *p* = 0.005; HIV, *p* = 0.048) and overall emotional distress (hepatitis, *p* = 0.020). Spearman's rank correlation further revealed significant associations between NRS, pain persistence, PPT and HJHS with all psychological outcomes, though most effect sizes were weak.

**Conclusion:**

These results provide further evidence for a poorer psychological profile in PwH compared to the healthy population. Particularly, pain‐related outcomes, but also joint degeneration and the presence of viral infections, are related to enhanced psychological symptoms.

## 1 Introduction

Musculoskeletal disorders, such as osteoarthritis or haemophilic arthropathy, are of immense concern as they are associated with a higher likelihood of developing psychological symptoms such as depressive episodes or anxiety [1]. Haemophilic arthropathy represents a manifestation of maladaptive articular changes due to repeated bleeding events in patients with haemophilia (PwH), mainly in the ankle, knee and elbow joints [2]. The association of haemophilia with an impaired psychological well‐being has long been recognized. A recent systematic review and meta‐analysis stated that PwH have a staggeringly higher prevalence of depressive symptoms (41.7%) and rate of depression diagnosis (14.5%) compared to the general population [3]. The prevalence rate appears to be interrelated with the severity of the disease, with severely affected PwH being at greater risk [4]. Anxiety has also been reported to be heightened in PwH [4]. This could be of great relevance as results of one study suggest that acute anxiety states due to stressful life events might indirectly promote the risk of haemorrhage by increased fibrinolytic activity [5]. Stress‐related symptoms in the context of haemophilia have so far primarily been investigated from the parents’ perspective of the respective patients [6].

Several risk factors for the development of psychological disorders have already been established in the general population, such as, amongst others, genetic predisposition [7], lower socioeconomic status [8], or chronic poor sleep quality [9]. In addition, factors like pain interference and low treatment adherence, which are more specific to these patients’ circumstances, are thought to increase the risk of developing psychological disorders in PwH [10, 11]. Given that pain and functional limitations due to recurrent bleeding events and consequently structural alterations within the musculoskeletal system are highly prevalent amongst PwH [2] and are associated with impaired quality of life [12], these pathophysiological factors must be taken into account with respect to the aetiology of psychological symptoms. Therefore, this study aimed to investigate the psychological state in PwH in comparison to a healthy control cohort using the Depression Anxiety and Stress Scale 21 (DASS). Further, it was aimed to explore the impact of inherent (e.g., type, severity) and acquired (e.g., pain, joint degeneration) disease‐specific factors on the patient's level of depression, anxiety and stress. By doing so, the range of potential influencing factors on the mental health of PwH can be expanded on more thoroughly.

### 1.1 Materials and Methods

#### 1.1.1 Subjects

In the present investigation, male patients with mild, moderate, or severe haemophilia A or B and age‐matched healthy controls were included based on their prior participation in previously completed or ongoing multicentre observational studies. For the purposes of this secondary analysis, eligible participants were consolidated into a single database after excluding potential duplicate entries and re‐evaluated in light of the modified research question posed in this study. Data were originally collected over a 5‐year period, from 2018 to 2023. All underlying studies were real‐world, non‐interventional trials in which standard care was not voluntarily altered (e.g., no treatment switches). This analysis was restricted to baseline data from the original studies. Additionally, data from healthy controls were universally derived from the same observational studies as the patient data and were specifically collected as comparative references for the respective patient group. Patients were recruited as part of the observational studies during their routine clinical assessments, while healthy controls were recruited either through the respective centres or via social media announcements and personal contacts. Patients with mild and moderate haemophilia are hereinafter classified as patients with non‐severe haemophilia. Subjects were considered eligible if they submitted informed consent and were at least 18 years of age. Individuals in both groups were excluded if one of the following exclusion criteria was satisfied: diagnosed bleeding disease apart from haemophilia, joint surgery (e.g., arthroscopy, synovectomy) within the last 6 months prior to examination or occurrence of a bleeding event 2 weeks before enrolment in the study. Moreover, healthy participants with a diagnosed musculoskeletal disorder (e.g., osteoarthritis), pain‐related disorder (e.g., fibromyalgia), or those with an acute musculoskeletal injury (e.g., ankle sprain) were excluded from participation. In addition, participants were asked about the use of analgesics. Regarding the multicentric design, data were collected by three different examiners (A.S., M.B., P.M.), all belonging to the same research group. Each of them received identical instructions on the standardized measurement methodologies and procedures.

#### 1.1.2 Questionnaire‐Based Outcomes

The DASS is a well‐established self‐report instrument that evaluates a patient's emotional state [13]. It represents one rubric of the German Pain Questionnaire, whose psychometric properties have been studied in a variety of nationalities and cultures with uniformly good results [14, 15, 16]. The questionnaire is divided into three distinct scales of depression (e.g., low mood levels, suicidal ideations), anxiety (e.g., fearful thoughts, expectation of negative events) and stress (e.g., continuous levels of autonomic arousal, decreased frustration tolerance). Each scale includes seven specific items, resulting in a total of 21 items, representing overall levels of emotional distress. Each item is based on a 4‐point Likert scale that requires a self‐assessment of each individual statement within the last week. The scale ranges from 0 (‘did not apply to me at all’) to 3 (‘applied to me very much or most of the time’). A higher score uniformly indicates a more negative emotional state.

Lastly, a specifically designed anamnestic questionnaire was distributed to collect personal and disease‐specific data such as type and severity of haemophilia, treatment modality, diagnosed comorbidities and surgical interventions.

#### 1.1.3 Pain‐Related Outcomes

PPTs were assessed utilizing a handheld pressure algometer (Wagner Instruments, Greenwich, Connecticut, USA). The measurement instrument, equipped with a rubberized end piece at the tip, was placed on the joint space of the subjectively most affected joint (PPT_aff_), as well as the contralateral side (PPT_contra_), as described elsewhere [17]. PPT measurements were exclusively taken at the ankle, knee and elbow joints. Subjects were instructed to report when the constantly increased pressure (10 Newton/second) first became painful. The mean value of three consecutive series of measurements was used for statistical analysis. According to the current literature, an acceptable to excellent inter‐rater reliability was observed for PPT in patients with knee osteoarthritis [18, 19]. Lower numerical values are defined as more enhanced levels of pain sensitivity.

In addition, general and not joint‐specific self‐perceived pain intensity was evaluated on two temporal scales, both on a numerical rating scale (NRS). Subjects were asked to rate their current pain intensity (NRS‐now) as well as their average pain intensity over the past 4 weeks (NRS‐4w) on a scale ranging from 0 (no pain) to 10 (maximum pain imaginable). Finally, pain persistence was reported by the participants on a 6‐point scale with different time‐dependent gradations. The predefined time periods for persistence of pain in order from 1 to 6 were as follows: ‘less than 1 month’, ‘1 month to half a year’, ‘half a year to 1 year’, ‘1 year to 2 years’, ‘2 years to 5 years’ and ‘more than 5 years’.

#### 1.1.4 Orthopaedic Joint Status

In the context of haemophilia, the validated Haemophilia Joint Health Score 2.1 (HJHS) is generally used to assess the structural and functional joint condition [20]. All three aforementioned joints were evaluated on the basis of multiple parameters (e.g., muscle atrophy, range of motion, swelling) to gauge the magnitude of arthropathic joint alterations. A maximum score of 20 points was assigned for each of the six joints, plus a gait analysis with a maximum score of 4 points, corresponding to a total score of 124 points. The higher the score, the more severely the joint status is impaired. As for PPT, excellent inter‐observer reliability exists for the HJHS [21].

#### 1.1.5 Statistics

Data were analytically and visually tested for normal distribution by Kolmogorov–Smirnov tests and Q‐Q plots, respectively. Person‐related (e.g., age, BMI) and arthropathy‐specific data (i.e., NRS‐now NRS‐4w, HJHS) were normally distributed and consequently statistically analysed by employing Student's *t*‐tests. Data on psychological outcomes violated normal distribution; hence, between‐group differences based on person‐related (i.e., age, BMI) and disease‐specific measures (i.e., type, severity, treatment, HIV, hepatitis) as well as correlation analyses were calculated by the employment of Mann–Whitney *U* tests and Spearman's rank correlations, respectively. Data on hepatitis was dichotomized (0 = not present, 1 = present) and therefore any type of hepatitis was clustered into one single group. To further identify potential mediation effects of age and BMI, covariate‐adjusted between‐group differences and Spearman's rank correlation analyses were used. Spearman's correlation coefficients and effect sizes based on Pearson's *r* were interpreted based on the generally accepted conventions formulated by Cohen: weak = 0.10–0.29, moderate = 0.30–0.49, strong ≥ 0.50 [22]. Statistical analyses were conducted via IBM SPSS 27 (Armonk, NY, USA) for Windows with a predefined alpha level of *p* ≤ 0.05.

## 2 Results

### 2.1 Subjects

Overall, 379 PwH and 271 healthy controls were enrolled and statistically analysed. The majority of patients with severe haemophilia (93.4%) were treated prophylactically, whereas approximately three‐quarters of patients with non‐severe haemophilia (76.1%) were receiving on‐demand treatment at the time of enrolment. Further, 29 PwH (7.7%) presented with viral coinfections (i.e., PwH with HIV and hepatitis). All patients with a history of hepatitis had previously completed successful treatment and did not experience any acute complications related to the condition during the study period. Subject characteristics of both study groups, as well as haemophilia‐related data of patients, are presented in Table 1.

**TABLE 1:** Subject characteristics and haemophilia‐specific data in patients with haemophilia (PwH, *n* = 379) and healthy controls (Con, *n* = 271).

Parameter	PwH	Con	*p* value
Age (years)	41.7 ± 15.8 (13.0–79.0)	37.2 ± 14.6 (13.0–80.0)	<0.001
Height (m)	1.80 ± 0.08 (1.53–2.00)	1.82 ± 0.07 (1.63–2.00)	0.071
Weight (kg)	84.7 ± 17.1 (40.0–170.0)	82.7 ± 10.8 (54.5–123.0)	<0.001
BMI (kg/m^2^)	26.0 ± 4.8 (17.1–49.7)	24.9 ± 3.0 (17.6–39.7)	<0.001
Type and severity	Severe A: 230 (61) Severe B: 36 (9) Moderate A: 55 (15) Moderate B: 14 (4) Mild A: 38 (10) Mild B: 6 (1)		
Treatment regimen^a^	Prophylaxis: 268 (72) On demand: 103 (28)		
Subjectively most affected joint^b^	None: 48 (13) Ankle left: 64 (18) Ankle right: 84 (23) Knee left: 46 (12) Knee right: 57 (16) Elbow left: 11 (3) Elbow right: 25 (7) Others: 30 (8)		
HIV	No: 332 (88) Yes: 47 (12)		
Hepatitis^c^	No: 311 (83) Yes: 65 (17)		
HJHS^[Link]^, ^[Link]^	20.1 ± 17.4 (0.0–94.0)	4.3 ± 3.8 (0.0–29.0)	<0.001
NRS‐now	2.1 ± 2.4 (0.0–10.0)	0.4 ± 1.0 (0.0–7.0)	<0.001
NRS‐4w	4.8 ± 3.1 (0.0–10.0)	1.7 ± 2.5 (0.0–9.0)	<0.001
Pain persistence^[Link]^, ^[Link]^	4.1 ± 2.0 (1.0–6.0)	3.4 ± 2.0 (1.0–6.0)	<0.001

*Note*: Data presented as mean ± standard deviation (min‐max) or absolute frequency (%). Differences are considered significant for *p* ≤ 0.05, employing Student's *t*‐test.

Abbreviations: BMI, body mass index; HJHS, Haemophilia Joint Health Score v2.1; NRS‐now, current general, not joint‐specific pain intensity; NRS‐4w, average general, not joint‐specific pain intensity across 4 weeks.

^a^ *n* = 371;.

^b^ *n* = 365;.

^c^ *n* = 376;.

^¥^ *n* = 377 (PwH),.

^¥^ *n* = 174 (Con),

^†^ *n* = 356 (PwH),

^†^ *n* = 251 (Con).

### 2.2 Disease‐Specific Factors and Its Association With Psychological Outcomes

Between‐group analyses revealed statistically significant differences for depression (*p* < 0.001), anxiety (*p* = 0.002), stress (*p* < 0.001) and DASS total score (*p* < 0.001), with PwH demonstrating worse outcomes across all parameters compared to the healthy cohort (Table 2). Further analyses for the patient group indicated no significant differences in any of the psychological indices in terms of body mass index, type of haemophilia, disease severity and treatment modality. However, PwH suffering from a hepatitis infection showed more severe signs of depression (*p* = 0.006, r = 0.120), higher stress levels (*p* = 0.027, r = 0.143) and higher overall emotional distress (*p* = 0.007, *r* = 0.119). Additionally, PwH older than 40 years (*p* = 0.026, *r* = 0.105) and patients infected with HIV (*p* = 0.048, *r* = 0.101) both demonstrated significantly higher scores for stress (Table 3).

**TABLE 2:** Depression, anxiety and stress scores based on the DASS 21 questionnaire in patients with haemophilia (PwH, *n* = 379) and healthy controls (Con, *n* = 271).

Parameter	PwH	Con	*p* value	Effect size
Depression	2.3 ± 3.3 [1.0] (0.0–18.0)	1.2 ± 2.3 [0.0] (0.0–15.0)	<0.001	0.197
Anxiety	1.4 ± 2.3 [0.0] (0.0–19.0)	0.9 ± 1.6 [0.0] (0.0–9.0)	0.002	0.123
Stress	3.3 ± 3.9 [2.0] (0.0–21.0)	2.1 ± 3.0 [1.0] (0.0–18.0)	<0.001	0.171
Total score	7.0 ± 8.5 [4.0] (0.0–57.0)	4.2 ± 6.0 [2.0] (0.0–33.0)	<0.001	0.188

*Note*: Data presented as mean ± standard deviation [median] (min‐max). Differences are considered significant for *p* ≤ 0.05, employing Mann–Whitney *U* test.

Abbreviation: DASS, Depression Anxiety and Stress Scale 21.

**TABLE 3:** Association between psychological outcome scales based on the DASS 21 questionnaire with anthropometric and disease‐specific parameters in patients with haemophilia (PwH).

Group		*N*	Depression	*p* *r*	Anxiety	*p* *r*	Stress	*p* *r*	Total score	*p* *r*
Age	≤40 >40	191 187	1.9 ± 2.8 [1.0] (0.0–17.0) 2.7 ± 3.8 [1.0] (0.0–18.0)	0.106 *0.071*	1.2 ± 2.1 [0.0] (0.0–19.0) 1.5 ± 2.4 [1.0] (0.0–13.0)	0.429 *0.003*	2.9 ± 3.5 [2.0] (0.0–21.0) 3.8 ± 4.2 [2.0] (0.0–18.0)	**0.026** *0.105*	6.0 ± 7.4 [4.0] (0.0–57.0) 8.0 ± 9.5 [5.0] (0.0–48.0)	0.131 0.077
BMI	<25 ≥25	175 204	2.2 ± 3.0 [1.0] (0.0–17.0) 2.4 ± 3.5 [1.0] (0.0–18.0)	0.647 *0.020*	1.2 ± 1.9 [0.0] (0.0–11.0) 1.5 ± 2.6 [1.0] (0.0–19.0)	0.852 *0.036*	3.5 ± 3.9 [2.0] (0.0–20.0) 3.2 ± 3.8 [2.0] (0.0–21.0)	0.453 *0.029*	6.9 ± 7.9 [4.0] (0.0–48.0) 7.1 ± 9.1 [4.5] (0.0–57.0)	0.736 *0.005*
Type	A B	323 56	2.4 ± 1.8 [1.0] (0.0–18.0) 1.8 ± 3.2 [1.0] (0.0–17.0)	0.089 *0.085*	1.3 ± 2.2 [0.0] (0.0–13.0) 1.6 ± 2.9 [1.0] (0.0–19.0)	0.591 *0.029*	3.3 ± 3.8 [2.0] (0.0–20.0) 3.3 ± 4.3 [2.0] (0.0–21.0)	0.631 *0.022*	7.1 ± 8.3 [4.0] (0.0–48.0) 6.7 ± 9.7 [4.0] (0.0–57.0)	0.382 *0.043*
Seve‐ Rity	S NS	262 112	2.4 ± 3.4 [1.0] (0.0–18.0) 2.2 ± 3.2 [1.0] (0.0–15.0)	0.277 *0.043*	1.4 ± 2.3 [0.0] (0.0–19.0) 1.4 ± 2.2 [0.5] (0.0–13.0)	0.906 *0.005*	3.4 ± 3.8 [2.0] (0.0–21.0) 3.2 ± 4.0 [2.0] (0.0–17.0)	0.237 *0.044*	7.2 ± 8.6 [5.0] (0.0–57.0) 6.8 ± 8.6 [4.0] (0.0–45.0)	0.390 *0.031*
Treat‐ment	Pro OD	268 103	2.4 ± 3.5 [1.0] (0.0–18.0) 2.0 ± 2.8 [1.0] (0.0–13.0)	0.243 *0.053*	1.4 ± 2.5 [0.0] (0.0–19.0) 1.2 ± 1.9 [0.0] (0.0–11.0)	0.841 *0.003*	3.5 ± 4.0 [2.0] (0.0–21.0) 3.0 ± 3.4 [2.0] (0.0–15.0)	0.430 *0.030*	7.3 ± 9.1 [4.0] (0.0–57.0) 6.2 ± 7.1 [4.0] (0.0–36.0)	0.560 *0.021*
HIV	No Yes	332 47	2.2 ± 3.1 [1.0] (0.0–17.0) 3.1 ± 4.5 [1.0] (0.0–18.0)	0.678 *0.046*	1.3 ± 2.2 [0.0] (0.0–19.0) 1.7 ± 2.7 [0.0] (0.0–13.0)	0.895 *0.022*	3.2 ± 3.8 [2.0] (0.0–21.0) 4.4 ± 4.4 [4.0] (0.0–18.0)	**0.048** *0.101*	6.7 ± 8.2 [4.0] (0.0–57.0) 9.1 ± 10.6 [7.0] (0.0–48.0)	0.495 *0.066*
HEP	No Yes	311 68	2.2 ± 3.3 [1.0] (0.0–18.0) 3.0 ± 3.4 [2.0] (0.0–14.0)	**0.006** *0.120*	1.3 ± 2.3 [0.0] (0.0–19.0) 1.6 ± 2.4 [1.0] (0.0–12.0)	0.061 *0.061*	3.1 ± 3.7 [2.0] (0.0–21.0) 4.5 ± 4.2 [4.0] (0.0–17.0)	**0.027** *0.143*	6.5 ± 8.4 [4.0] (0.0–57.0) 9.1 ± 9.0 [7.0] (0.0–36.0)	**0.007** *0.119*
HIV and HEP	No Yes	350 29	2.1 ± 3.0 [1.0] (0.0–11.0) 2.3 ± 3.3 [1.0] (0.0–18.0)	0.679 *0.080*	1.1 ± 2.0 [0.0] (0.0–8.0) 1.4 ± 2.4 [0.0] (0.0–19.0)	0.487 *0.134*	3.5 ± 3.7 [2.0] (0.0–12.0) 3.3 ± 3.9 [2.0] (0.0–21.0)	0.822 *0.044*	6.6 ± 7.7 [4.0] (0.0–30.0) 7.0 ± 8.6 [4.0] (0.0–57.0)	0.806 *0.047*

*Note*: Data presented as mean ± standard deviation [median] (min‐max). Differences are considered significant for *p* ≤ 0.05, employing age‐ and BMI‐adjusted non‐parametric analyses. Effect sizes are presented as Pearson's *r* and are presented in italics, while significant differences are highlighted in bold.

Abbreviations: BMI, body mass index; DASS, Depression Anxiety and Stress Scale 21; HEP, hepatitis; NS, non‐severe; OD, on demand; Pro, prophylaxis; S, severe; type, type of haemophilia.

### 2.3 Arthropathy‐Related Factors and Its Association With Psychological Outcomes

A total of 62% of PwH experienced pain (NRS‐now ≥ 1) at the time of data collection, and 85% reported pain to be present in the past 4 weeks (NRS‐4w ≥ 1) compared to 19% and 41% of healthy controls, respectively. Pain persisting for more than 2 years was evident in 48% of PwH and significantly more prolonged compared to healthy controls (*p* < 0.001). Pain‐related parameters showed significant correlations with any of the psychological outcomes, according to correlation analyses adjusted for age and BMI (*p* ≤ 0.05), except for the association of PPT_contra_ and stress (unadjusted: *r*_s_ = −0.105; *p* = 0.105; adjusted for age: r_s_ = −0.108; *p* = 0.097; adjusted for BMI: *r*_s_ = −0.104; *p* = 0.107). When adjusting the correlation between HJHS and anxiety for age, the statistically significant association could not be preserved (*r*_s_ = 0.093; *p* = 0.071). An overview of unadjusted and covariate‐adjusted correlation analyses can be obtained in Table 4.

**TABLE 4:** Unadjusted and covariate‐adjusted (control variables: Age and BMI) Spearman correlations between psychological outcome scales based on the DASS 21 questionnaire and pain, as well as joint score with haemophilia (PwH).

Parameter	Condition	*N*	Depression r_s_ (*p* value)	Anxiety r_s_ (*p* value)	Stress r_s_ (*p* value)	Total score r_s_ (*p* value)
NRS‐now	Unadjusted Age BMI	379 378 379	0.258 (<0.001) 0.246 (<0.001) 0.262 (<0.001)	0.168 (<0.001) 0.156 (0.002) 0.164 (<0.001)	0.261 (<0.001) 0.232 (<0.001) 0.274 (<0.001)	0.264 (<0.001) 0.249 (<0.001) 0.273 (<0.001)
NRS‐4w	Unadjusted Age BMI	378 377 378	0.316 (<0.001) 0.306 (<0.001) 0.320 (<0.001)	0.234 (<0.001) 0.227 (<0.001) 0.233 (<0.001)	0.299 (<0.001) 0.279 (<0.001) 0.311 (<0.001)	0.326 (<0.001) 0.314 (<0.001) 0.334 (<0.001)
PPT_aff_	Unadjusted Age BMI	240 239 240	−0.208 (<0.001) −0.214 (<0.001) −0.207 (<0.001)	−0.196 (0.002) −0.201 (0.002) −0.195 (0.002)	−0.170 (0.008) −0.180 (0.005) −0.170 (0.008)	−0.213 (<0.001) −0.221 (<0.001) −0.213 (<0.001)
PPT_contra_	Unadjusted Age BMI	241 240 241	−0.192 (0.003) −0.194 (0.003) −0.183 (0.003)	−0.155 (0.016) −0.157 (0.015) −0.157 (0.015)	−0.105 (0.105) −0.108 (0.097) −0.104 (0.107)	−0.153 (0.018) −0.155 (0.016) −0.153 (0.018)
Pain persistence	Unadjusted Age BMI	356 355 356	0.286 (<0.001) 0.275 (<0.001) 0.285 (<0.001)	0.242 (<0.001) 0.234 (<0.001) 0.240 (<0.001)	0.272 (<0.001) 0.252 (<0.001) 0.275 (<0.001)	0.309 (<0.001) 0.296 (<0.001) 0.309 (<0.001)
HJHS	Unadjusted Age BMI	377 377 377	0.163 (0.002) 0.140 (0.006) 0.161 (0.002)	0.111 (0.031) 0.093 (0.071) 0.107 (0.039)	0.183 (<0.001) 0.139 (0.007) 0.187 (<0.001)	0.178 (<0.001) 0.153 (0.003) 0.179 (<0.001)

Abbreviations: DASS, Depression Anxiety and Stress Scale 21; HJHS, haemophilia joint health score; NRS‐4w, average general, not joint‐specific pain across the last 4 weeks on a numeric rating scale; NRS‐now, current general, not joint‐specific pain on a numeric rating scale; PPTcontra, pressure pain thresholds of the contralateral joint; PPTmaj, pressure pain thresholds of the subjectively most affected joint; r_s_, Spearman's coefficient.

## 3 Discussion

This study aimed to investigate the presence of psychological symptoms in PwH in comparison to the healthy population and to explore the potential impact of disease‐specific and arthropathy‐related factors inherent to haemophilia on the psychological profile using the DASS. Results revealed that PwH uniformly demonstrate increased scores for depression, anxiety and stress compared to healthy individuals. Furthermore, disease severity, type of haemophilia and treatment regimen do not appear to have a significant influence on psychological symptoms, while the presence of concomitant viral infections such as HIV and hepatitis in PwH might influence the psychological profile. Moreover, data suggest an association between depression, anxiety, stress and overall emotional distress with pain persistence, pain intensity, PPT_aff_ and PPT_contra,_ as well as the structural and functional joint status.

Psychological well‐being in PwH has long been of interest to researchers, and a worse profile compared to the general population has been documented descriptively and analytically in various studies [3, 23]. Witkop et al. [24] reported that, based on self‐report, a substantial number of PwH meet the criteria for depression (30%), anxiety (25%) and stress (27%). Given the demonstrated differences in BMI and age between PwH and healthy controls, one can presume that those factors might in part be responsible for the higher scores of psychological symptoms in PwH. In particular, BMI seems relevant, considering the well‐established association with depression [25, 26]. However, when controlling for both variables via partial correlation analyses (see Table 4), with the exception of the association between HJHS and anxiety, no significant deviations were observed from the uncontrolled analyses, suggesting that age and BMI do not substantially mediate the relationships of interest. Consequently, the hypothesis emerges that distinct factors specific to haemophilia and arthropathy, such as disease severity, pain intensity, pain persistence and in part functional deficits, might contribute to this notion.

In the last decades, pain has repeatedly been linked to the development of psychological symptoms in PwH, and a general consensus exists on their bidirectional interplay [26, 27]. This finding is consistent with other musculoskeletal disorders like osteoarthritis [28]. In line with these results, the presented data suggest increased levels of depressive and anxiety symptoms in patients with higher self‐reported pain intensity and pain persistence. Additionally, PPT_aff_ as well as PPT_contra_ demonstrated significant inverse correlations with psychological measures, which have not yet been analysed. This translates into an enhanced pain sensitivity towards mechanical stimuli, the more severe the psychological symptoms. Interestingly, not just the subjectively most affected joint showed decreased PPT values, but also the contralateral joint. This implies that the link between psychological measures and pain sensitivity is more general in nature. Pain signalling is therefore altered on a more global scale and is not restricted to a patient's subjective perception of their joint status. Though it should be noted that the majority of the presented correlation analyses yielded coefficients that are to be interpreted as weak.

In terms of joint status based on HJHS, a positive low linear correlation with depression, anxiety, stress and overall emotional distress was observed. This finding suggests that PwH with a worse joint status show higher levels of psychological symptoms. This association was previously demonstrated by Pinto et al. [10], but using subjective measures of an individual's joint health. Therefore, both subjectively perceived and objectively assessed joint health might predict worse outcomes in terms of psychological well‐being, which makes intuitive sense considering the musculoskeletal limitations haemophilic arthropathy might impose on daily living and quality of life [29].

According to our data, patients with HIV exhibit higher stress scores, while those with viral hepatitis show increased scores for depression, stress and overall emotional distress, even after adjusting for age and BMI. This finding is particularly noteworthy in relation to patients with hepatitis, as all individuals in this subgroup had previously undergone successful treatment for the infection. This observation is in concordance with studies exploring the association between viral infections and depression and stress, suggesting that the existence of viral infections might predispose patients to more severe psychological consequences [30, 31, 32]. The results may be attributed to the chronic nature of these infections, the associated stigma and the ongoing uncertainty about disease progression, all of which can contribute to elevated emotional distress [33]. It should be noted, however, that the observed effect sizes were small and should be interpreted with caution. Interestingly, no significant differences were observed between PwH with coexisting viral infections (HIV and hepatitis) and those with either one or no viral infection. This could suggest that the presence of a single chronic viral infection may already be sufficient to trigger psychological symptoms and that additional infections do not necessarily compound the effect significantly. Moreover, PwH living with coexisting chronic coinfections may develop coping mechanisms over time that help mitigate psychological burden, potentially balancing out group differences. In this context, the time since diagnosis may play an important role, as effective coping strategies typically require time and external support to develop [34, 35]. However, as no data on the time point of diagnosis were available, this relationship could not be determined and warrants further investigation. Contrastingly, parameters specific to haemophilia (e.g., type, severity, treatment modality) did not show significant associations with psychological outcomes. This distinction could possibly be explained by the fact that patients with HIV or hepatitis also had significantly higher values for pain intensity, pain persistence, or HJHS compared to patients without the respective viral infection. A significant difference in HJHS and pain persistence was also found for PwH with different disease severities and treatment regimens. For pain intensity, however, discrepancies could only be observed for subgroups regarding viral infections, suggesting that perceived pain intensity may indirectly mediate the relation between viral comorbidities and worse psychological outcomes in PwH. Given the non‐normal distribution of residuals, a hierarchical regression model could not be applied to statistically examine overarching predictors of psychological outcomes. This was done in a recent study by Pinto and colleagues [10], who found pain intensity to be positively predictive of anxiety‐related as well as depressive symptoms using the PROMIS questionnaire. However, pain interference and not pain intensity was most strongly associated with both psychological domains. Considering the high prevalence of chronic pain amongst PwH and its strong association with symptoms of depression, anxiety and stress, future research efforts should continue to better understand and ameliorate pain and, hence, improve the psychological profile. Furthermore, it should be considered that additional factors not assessed in the present study, such as lifestyle‐related variables (e.g., poor sleep quality, low levels of physical activity) and specific disease‐related concerns (e.g., fear of bleeding episodes, regular intake of pain medication, loss of educational/occupational opportunities), may also contribute to the heightened psychological burden observed in PwH. These factors should be explored in future studies using multivariate models alongside those factors identified in the present investigation.

To tackle the heightened psychological symptoms in PwH, psychology‐based interventions have increasingly been evaluated in the past years. Unfortunately, according to a systematic Cochrane review by Palareti et al. [36], mood‐related outcomes have sparsely been investigated and the results yield a high degree of uncertainty due to methodological heterogeneity. The authors point out that higher methodological consistency and quality is needed to draw meaningful conclusions from these trials. More recently, numerous studies looked at subjective pain and health‐related quality of life as primary outcomes after employment of a psychology‐based intervention. In the majority of investigations, both parameters were significantly improved as a result of hypnosis or breath‐manipulation protocols [37, 38]. However, the efficacy of those practices on pain sensitivity in PwH has not been investigated yet. Future research should therefore aim to explore whether similar results could be observed for experimentally induced pain. Nonetheless, according to the current guidelines for haemophilia treatment, patients should be educated on the beneficial effects of psychological interventions and implement these in daily life on a regular basis [39].

### 3.1 Strengths and Limitations

This study provides data on a clinically relevant topic within a large sample size of patients suffering from the rare disease haemophilia and healthy individuals. Furthermore, pain sensitivity via PPTs was, for the first time, linked to psychological symptoms in PwH. However, this study also has some limitations worth mentioning. First, assessment of psychological symptoms was done by self‐report and not per diagnosis of a board‐certified healthcare provider. Therefore, no clinically verified prevalence rates of psychological disorders could be calculated and presented. Second, the initially intended analysis of the main determinants using hierarchical regression models could not be realized due to a violation of data normality. Third, data on other relevant and potential confounders such as physical activity levels, pain interference, socioeconomic status, presence of target joints, choice of treatment regimen (e.g., factor replacement, non‐factor therapy) and sleep quality were not assessed and could therefore not be analysed. Fourth, the assessment of subjective pain was restricted to global and not joint‐specific pain, limiting the ability to directly relate PPT measurements to localized chronic pain at specific joints. Fifth, as the data were derived solely from observational studies, which might be prone to certain biases (e.g., selection bias, collider bias), this limitation should be considered when interpreting the results, as they may not reflect causal relationships. Lastly, a significant difference in age was found between PwH and healthy controls, which in theory could be responsible for the differences observed in psychological metrics. However, individuals in both groups are, on average, in the same stage of life and thus face comparable challenges in their daily lives on average. This suggests that, looking at the between‐group comparisons of psychological symptoms, it may not lead to a significant influence of age.

## 4 Conclusion

The results presented herein provide further evidence for a poorer psychological profile of PwH compared to the healthy population. Particularly, pain‐related parameters, but also manifested arthropathy and the presence of viral infections, might increase the susceptibility to feelings of depression, anxiety, stress and overall emotional distress. Furthermore, the data indicate an inverse association between psychological symptoms and pain sensitivity as measured by PPT. As arthropathy‐related factors such as pain and joint deterioration appear to affect psychological well‐being, prevention of haemophilic arthropathy is of great importance, also to maintain good quality of life. Given the uniformly weak effect sizes for significant differences, the presented results should be interpreted with caution and further analyses are needed to support these findings.

## Author Contributions

Alexander Schmidt, Fabian Tomschi and Thomas Hilberg designed the study. Alexander Schmidt, Fabian Tomschi, Pia Möllers and Marius Brühl performed the data collection, and Alexander Schmidt performed the data analysis. Andreas Christian Strauss, Heinrich Richter and Johannes Oldenburg supported the recruitment process and provided scientific expertise together with Fabian Tomschi and Thomas Hilberg. Alexander Schmidt, Fabian Tomschi and Thomas Hilberg drafted the manuscript. Thomas Hilberg additionally supervised the project.

## Ethics Statement

This study was approved by the local Ethical Review Boards (University of Wuppertal MS/BB 180613; Westphalia Lippe Medical Association: 2018‐510‐f‐S; University Hospital Bonn: 339/19 and 329/19).

## Conflicts of Interest

A. Schmidt has received speakers’ fees and travel expenses from Sobi and Takeda. F.T. has received speakers’ fees from Takeda and received an educational grant from Sobi. P.R. has received speakers’ fees and travel support from Takeda as well as travel support from Sobi. M.B. has received travel fees from Sobi and Takeda. H.R. has received research and travel grants from Bayer, Biotest, CSL Behring, Intersero, Novo Nordisk, Pfizer, Sobi and Takeda. J.O. has received research funding from Bayer, Biotest, CSL Behring, Octopharma, Pfizer, Sobi and Takeda as well as honoraria (for consultancy, speakers’ bureau, scientific advisory board) and/or travel expenses from Bayer, Biogen Idec, Biomarin, Biotest, Chugai, CSL Behring, Freeline, Grifols, LFB, Novo Nordisk, Octopharma, Pfizer, Roche, Sanofi, Spark Therapeutics, Sobi and Takeda. A. C. Strauss has received research funding from Bayer, Sobi and Takeda and has received consultancy, speakers’ bureau, honoraria, scientific advisory board and travel expenses from Bayer, Biotest, CSL Behring, Novo Nordisk, Sobi and Takeda. T.H. has received research funding from Biotest, Chugai, CSL Behring, Intersero, Roche, Sobi and Takeda as well as travel expenses, speakers’ or scientific advisory board honoraria from Bayer, Biotest, Chugai, Novo Nordisk, Pfizer, Roche, Sanofi, Sobi and Takeda.

## Data Availability

The data that was used for analysing the results provided are available from the corresponding author upon reasonable request.
